# What makes health impact assessments successful? Factors contributing to effectiveness in Australia and New Zealand

**DOI:** 10.1186/s12889-015-2319-8

**Published:** 2015-10-03

**Authors:** Fiona Haigh, Elizabeth Harris, Ben Harris-Roxas, Fran Baum, Andrew L. Dannenberg, Mark F. Harris, Helen Keleher, Lynn Kemp, Richard Morgan, Harrison NG Chok, Jeff Spickett

**Affiliations:** Centre for Health Equity Training, Research and Evaluation CHETRE, Ingham Institute, University of New South Wales, Sydney, 2052 Australia; Centre for Primary Health Care and Equity, University of New South Wales, Sydney, 2052 Australia; Southgate Institute for Health, Society & Equity, Flinders University, Adelaide, Australia; University of Washington School of Public Health, Seattle, USA; School of Public Health and Preventive Medicine, Monash University, Melbourne, Australia; Centre for Impact Assessment Research and Training (CIART), Department of Geography, University of Otago, Dunedin, New Zealand; WHO Collaborating Centre in Environmental Health Impact Assessment and School of Public Health, Curtin University, Bentley, Australia

**Keywords:** Health impact assessment, Effectiveness, Australia, New Zealand, Case studies

## Abstract

**Background:**

While many guidelines explain how to conduct Health Impact Assessments (HIAs), less is known about the factors that determine the extent to which HIAs affect health considerations in the decision making process. We investigated which factors are associated with increased or reduced effectiveness of HIAs in changing decisions and in the implementation of policies, programs or projects. This study builds on and tests the Harris and Harris-Roxas’ conceptual framework for evaluating HIA effectiveness, which emphasises context, process and output as key domains.

**Methods:**

We reviewed 55 HIA reports in Australia and New Zealand from 2005 to 2009 and conducted surveys and interviews for 48 of these HIAs. Eleven detailed case studies were undertaken using document review and stakeholder interviews. Case study participants were selected through purposeful and snowball sampling. The data were analysed by thematic content analysis. Findings were synthesised and mapped against the conceptual framework. A stakeholder forum was utilised to test face validity and practical adequacy of the findings.

**Results:**

We found that some features of HIA are essential, such as the stepwise but flexible process, and evidence based approach. Non-essential features that can enhance the impact of HIAs include capacity and experience; ‘right person right level’; involvement of decision-makers and communities; and relationships and partnerships. There are contextual factors outside of HIA such as fit with planning and decision making context, broader global context and unanticipated events, and shared values and goals that may influence a HIA. Crosscutting factors include proactive positioning, and time and timeliness. These all operate within complex open systems, involving multiple decision-makers, levels of decision-making, and points of influence. The Harris and Harris-Roxas framework was generally supported.

**Conclusion:**

We have confirmed previously identified factors influencing effectiveness of HIA and identified new factors such as proactive positioning. Our findings challenge some presumptions about ‘right’ timing for HIA and the rationality and linearity of decision-making processes. The influence of right timing on decision making needs to be seen within the context of other factors such as proactive positioning. This research can help HIA practitioners and researchers understand and identify what can be enhanced within the HIA process. Practitioners can adapt the flexible HIA process to accommodate the external contextual factors identified in this report.

**Electronic supplementary material:**

The online version of this article (doi:10.1186/s12889-015-2319-8) contains supplementary material, which is available to authorized users.

## Background

Health Impact Assessment (HIA) is a tool designed to produce evidence-based recommendations to prospectively inform decision-making about proposed projects, plans, programs and policies in order to maximise their positive and minimise their negative impacts on health. HIA has evolved over the past 20 years from its origins in environmental impact assessment and Healthy Public Policy. It emphasises the need to define health broadly—incorporating consideration of a range of social, environmental and economic factors that determine health outcomes—and has been promoted as a tool to promote health equity [1–8].

A small but growing body of research has demonstrated the direct and indirect effectiveness of HIAs (see additional file 1). HIAs are often directly effective in changing, influencing and broadening the areas that will be considered by a program, policy or project due to the likely impact on health, and in some cases they have an immediate impact on health determinants. Even when HIAs are reported to have no direct effect on a decision they are often still effective in influencing decision-making processes [9–13]. In our study participants considered ‘effectiveness’ as being much broader than merely the direct impact of an HIA on decisions. Many noted changes in relationships, a better understanding of the determinants of health, and positive working relationships as major and sustainable impacts stemming from their involvement in HIAs [9, 11].

### Defining effectiveness in relation to HIA

At one level determining the effectiveness of a HIA is simple: it is the extent to which the HIA succeeds in bringing about the desired changes to decision-making and implementation. However difficulty in determining its effectiveness arises when there are differing expectations and understandings about what constitutes ‘success’ and what constitutes a ‘desired change’ [14]. There is no consensus on what success or desired changes should look like in all cases, largely because there are different understandings about the purpose and goals of HIAs [15]; different stakeholders have different expectations as to what a HIA should achieve. As a result there is currently no straightforward way to evaluate the effectiveness of HIAs.

HIA literature and training materials often conceptualise the HIA process as rational and deterministic where inputs produce linear, predictable changes in outputs; decision-makers obtain information, consider the pros and cons, make a decision and act accordingly [2, 5, 16]. HIA shares this simplistic conceptualisation with standard public health planning models which are often presented: with “an objective epistemology, an assumption that planning and implementation are two separate linear sequential activities, and an assumption that social systems change can be predicted and controlled” [17]. There are assumptions about conscious control over these decisions, linearity, predictability, defined decision points and decision-makers. We anticipated at the start of this research that the reality of HIA practice and the decision processes HIAs attempt to influence may not reflect a linear model.

There are numerous guidelines and training courses now available explaining how to conduct HIAs [2]. However, a gap in research exists concerning the factors that influence the effectiveness of HIAs: that is, what makes some HIAs successful and others less so? Whilst some case study-based evaluations have been conducted, which help identify factors influencing effectiveness [7, 18], there is limited synthesis across multiple HIAs.

**Fig. 1:**
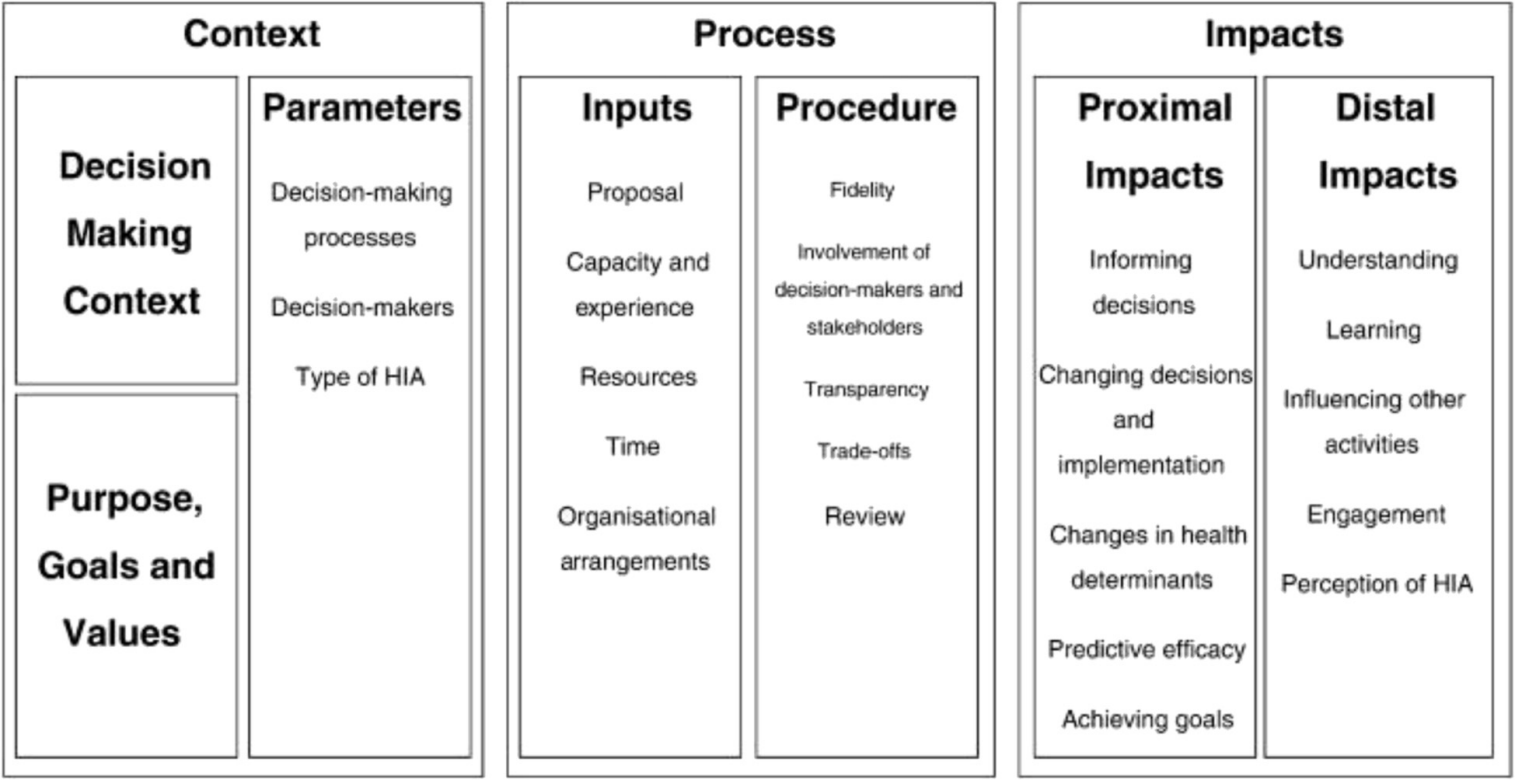
Original conceptual framework for the impact and effectiveness of health impact assessment

Harris-Roxas and Harris developed a conceptual framework for evaluating the effectiveness of a HIA that attempts to capture the broad range of factors that can determine whether a HIA is effective across decision-making and impact assessment contexts [19]. Harris-Roxas has gone on to review and modify the original conceptual framework [16] adopted for use in relation to equity focused health impact assessment. The modifications include adding timeliness as a process factor and timing as a contextual factor. Individual agency was also added as a context factor, reflecting the extent to which participants had a choice in participating in the process. A number of factors in the original framework were not found to be salient in their study. These included trade offs and review, predictive efficacy and achieving goals. The framework emphasises context, process and output as key domains, and has been used [6, 12, 16, 20, 21] and refined [16] as HIA practitioners seek to document effectiveness of their work. The original conceptual framework is shown in Fig. 1.

Our study is informed by the recent paper by Harris-Roxas et al. and builds on existing work in an attempt to determine factors influencing HIA effectiveness, by empirically testing the conceptual framework and identifying factors associated with increased or reduced HIA effectiveness [16]. This paper has two purposes. First we identify factors associated with increased or reduced effectiveness of HIAs in changing decisions and in the implementation of policies, programs or projects. We then test the conceptual framework proposed by Harris-Roxas to see whether it is consistent with our findings.

This is the first systematic, empirical study of the influence of HIA on decision-making and implementation in Australia and New Zealand [9–11]. To date most other studies on the effectiveness of HIA have relied on reviews of HIA documentation, except for Bourcier et al. which included interviews of significant stakeholders [22]. (Additional file 1 outlines previous studies into the effectiveness of HIAs). By contrast this study examined issues more deeply through interviews and triangulation of data. Interviews were conducted with:

(i) key decision-makers who were responsible for taking the recommendations forward
(ii) practitioners who conducted HIAs and
(iii) other stakeholders (including community stakeholders) involved in the process.

This gave us insight into multiple perspectives on how and why HIAs influence decisions and the actors involved.

In this paper we have examined in depth the factors that account for the extent, degree and sustainability of influence of HIAs across cases and context. We acknowledge that there is no simple recipe for effectiveness, but those factors closely associated with effectiveness give direction for practitioners and researchers in maximising the likelihood of success.

## Methods

The research used multiple methods for gathering and analysing qualitative and quantitative data, including: identification and mapping, survey and structured interviews and retrospective multiple case studies using qualitative methods [23, 24]. A four-phase process was used (Table 1). The research methods are described in more detail in previous publications [9, 10]. Ethics approval was given by the UNSW Human Research Ethics Committee (23 April 2010). Written consent forms provided information about the project, purpose of the interview, conditions of consent including anonymity and contact details. This paper mainly draws on the qualitative findings of phases 2, 3 and 4 with a particular focus on the case studies carried out in phase 3.

**Table 1:** Study phases and methods used in Australia/NZ HIA effectiveness study, 2005-2009

Phase 1 (*n* = 55 HIAs)	Identification and review: 55 Australian and NZ HIAs conducted during the period 2005–2009 were identified, characterised and reviewed using a validated review package [34] to determine the quality of the HIA reports [11].
Phase 2 (*n* = 48 HIAs)	Survey and interviews: Information was collected from the practitioners who conducted the HIAs, using a 29 item questionnaire and follow up interviews [11]. The questionnaire included a mix of open and closed questions that focused on their experiences and views on three aspects of their HIAs: process, context and decision making. We obtained completed questionnaires that covered 48 (87 %) of the 55 HIAs. We carried out 34 follow-up interviews, which covered 42 HIAs.
Phase 3 (*n* = 11 HIAs)	Case studies: Meta-evaluation of 11 case studies involving key informant interviews (*n* = 33) and document analysis. This allowed for developing a more in-depth understanding of HIA processes, studying complex systems and identifying contextual factors. We interviewed on average three people from each case study.
Phase 4	Integrative evaluation: The research team (*n* = 12 persons) carried out final analysis and evaluation of the research data over a three-day meeting followed by stakeholder validation workshop (*n* = 77 persons). This included triangulating the data from phases 1–3 to identify convergence, corroboration and correspondence of results from different methods and sources [35].

## Results

We found that the factors that expanded or refined the understanding of ways to influence effectiveness of HIAs could be broadly grouped into the Harris-Roxas model headings: Process related factors that include factors essential to HIA (necessary) and other process related factors that are not essential (contingent) but can enhance the impact of HIAs and broader contextual factors outside of the HIA process (see Table 2).

**Table 2:** Factors influencing effectiveness in Australia and New Zealand

HIA Process		Context
Necessary	Contingent	
Purposeful and structured stepwise process	Capacity and experience (right person and right level)	Fit with planning and decision making context
Flexibility	Involvement of decision-makers	Broader global context
Use of evidence	Involvement of communities	Unanticipated events and activities that may influence a HIA
	Relationships/Partnerships (organisational and individual level)	Shared values and goals
Cross cutting		
Time and timeliness		
Proactive positioning		

### Process factors

There are processes that are inherent to HIA that some participants felt made it a more effective tool than other inter-sectoral processes in which they had previously been involved. These include the stepwise process, the flexibility of HIA for adaptation to local contexts, and the legitimacy provided by evidence. Each of these features is discussed below.

#### Purposeful and structured stepwise process

The stepwise process was identified as a key strength of HIA because it made meetings and engagement in the process purposeful and structured. Respondents described how having meetings associated with the key steps of HIA (screening, scoping, assessment etc.) differed from ‘normal’ meetings in that there was a clear purpose to the meetings and that the meetings reflected progress being made in the HIA process. The structured ‘scientific’ process was also seen as creating or enhancing legitimacy. Having recommendations as an outcome of the process was also identified as being important.

*“Before …the people are in there but you’re not working together, you’re just providing your input and going “oh yeah whatever” … But then this way it was actually a combined effort… doing this [HIA] helped work out how to bring the cross divisional multiple professions together to work on a document that really was all about delivering sustainable communities”. (HIA working group, Engineering)*

#### Flexibility

The flexibility of the HIA allowed the stepwise process to be adapted to local contexts, to be culturally appropriate and to adjust to changes. Engagement with communities and cultural appropriateness was a particular feature of the New Zealand HIAs. Some HIAs utilised an HIA framework (Whanau Ora) designed specifically to be used on proposals that were likely to affect Maori health. The flexibility of HIA also allowed participants to adapt the process to changing circumstances. In some cases HIAs would be re-scoped part of the way through the process. For example, a decision that the HIA was meant to inform may have been made earlier than expected, and the HIA would then be re-scoped to focus on another facet of the proposal that could be influenced (e.g., implementation).

*“They worked the process so that they fit in the aspirations and dreams of the people. That doesn’t often happen. Too often you get to a certain place within the bureaucracy and then it stops because they say we’re not mandated to go that far, personally, they came to our Marae, and honoured that Marae and they were culturally sensitive, culturally appropriate, and so they did all the right things”. (Community leader)*

#### Use of evidence

Terms such as ‘structured’, ‘scientific’, ‘independent’ and ‘evidence-based’ were used to describe how HIA created or enhanced the legitimacy of having health considerations included in decision-making.

“… *that’s like science. You put it up there for public scrutiny and they can knock it down if they want to or they can support it. When it’s just your thoughts it’s not there for public scrutiny in the same sense as what a document like that is… The important thing is that you’re able to document and provide justification for the methods used and providing that you can do that, then your findings have some substance” (HIA Working Group, planner statutory agency)*

Other process related factors were identified that are not essential but can enhance the impact of HIAs. These factors related to which and how stakeholders are involved in the process.

There are often multiple individuals within the HIA process who are identified as having important roles in influencing the effectiveness of the HIA such as the right person at the right level, policy entrepreneur, the doer, decision-makers, community members, the HIA champion, and the problem maker. The individuals involved in a HIA have significant influence on both the process and outcomes of an HIA. Involvement of stakeholders including decision makers, members of potentially affected communities, and representatives of relevant stakeholder sectors (e.g. health, transport, planning) in carrying out the HIA was identified as being an important influence on HIA effectiveness.

#### Capacity and experience

Essential stakeholders are those who have the power to either make or influence decisions. To ensure the effectiveness of a HIA, we identified two main facets to this:

(i) having the direct involvement of the ‘right people’ and
(ii) ensuring that those people are at the ‘right level’ to be able to act on the findings of the HIA.

The right people are often at senior management level. They have some power (but not final decision making power), they understand the system well, often have pre-existing relationships that they can utilise and are in a position to influence the implementation of recommendations.

*“… one of the really critical things around keeping partnerships together is that you’ve got to have someone that’s got the delegation to keep it running …[because a] junior person couldn’t make decisions whereas, [name]and I and [name] can make decisions that enhances sustainability of it into the future” (HIA Working Group, Health Sector)*

In some case studies understanding the local context involved tactically bypassing the formal level of decision-making to a certain degree to enhance effectiveness.

*“If we had have gone to a general manager and said “Look we want to set up a partnership [to carry out a HIA]” maybe then they would say “No don’t worry, that’s not core business”. So we didn’t do it that way. We went through almost the back door and got it…” (decision maker and HIA commissioner, housing sector)*

Effectiveness is also related to the particular team of people brought together, not just the individual. Often in an effective HIA at least one member of the assessment team is skilled in engaging people in the process. For example, the lead in one HIA carefully selected the steering group members, made sure the meetings were well organised and then rang up stakeholders individually after the meetings to ensure the smooth running of the project.

*“[She] has been quite instrumental in a whole range of different areas. There’s been one in terms of just being able to build partnerships, get people on the side in her own quiet way and very skilful… you’ve got to have someone who’s keen on driving it so that the rest of you either come along or can check in and say okay where are we at”. (HIA working group, planner, government department)*

Key participants can act as HIA champions (advocates for HIA). For example, a senior manager in a council advocated for a HIA, which contributed to getting buy-in from the council. In two other HIAs, local councillors who had been involved in the HIA process went on to advocate for HIAs or to refer to the HIA when engaging in council business (e.g. at council meetings).

Respondents reported that involving decision-makers had a strong influence on effectiveness. Direct involvement appeared to be most powerful when the decision-making organisation was involved in the HIA working group (as opposed to steering group) and involvement in the assessment and recommendation stages was reported to be particularly important. Involving decision-makers directly in the process enabled HIA recommendations to be constructed in ways that would make them easier to adopt.

*“So that was actually really good, to have a councillor on there who'll be able to say, ‘that word itself [good neighbourliness] will be a block. Don't lose the concept but let’s think of a different way of presenting it.’” (HIA Working Group, Planning department)*

Directly involving decision makers in the process was also seen to lead to decision makers developing a feeling of ownership and responsibility for the HIA findings.

*“The benefits …. from my perspective is that by being quite involved in the preparation of the HIA, or certainly the business end of it from the consultation onwards, I feel a sense of ownership and obligation to try and keep that going.” (HIA working group, decision maker, local council)*

#### Relationships/Partnerships

Intersectorial involvement was seen to lead to relationship building between sectors, and the HIA process was seen to facilitate this*.* A participant commented how HIA:

*“is a legitimising mechanism for intersectorial working… this provided a platform to put those together in a cohesive way [and provided a] mechanism to legitimise why some people should be worried about other people’s work” (HIA working group, social planner)*

Intersectorial involvement was also seen to improve the quality of the HIA process and findings. In describing why the process worked, another stakeholder commented:

*“I think it was a good working team so you had a broad range of professions working together. So you had health professionals, community, engineering and I think we all learnt from each other. So you had some that would have pie in the sky ideas and others –you know me – keeps right on, follow the facts step by step, what are you going to do, how are you going to do it and so I think that bought it together to make that happen which was good”. (HIA working group, health sector)*

Our findings suggest that HIA legitimates, strengthens and increases the credibility of intersectoral processes and the processes themselves add legitimacy to HIA. Thus this creates a virtuous circle whereby each event increases the beneficial effect of the next [25].

#### Community involvement

Community involvement was also seen to be an important factor influencing effectiveness. Participants talked about how community involvement influences effectiveness. For example a participant described how input from the community changed the decision makers thinking about what the best location for a health service was - going against standard practice. They then went on to describe how the community input legitimised the change in decision.

*For me, on reflection, it was the best thing that ever happened because [key stakeholder] actually ended up being told through a process that involved community input that in fact, we made a decision that this is what the community wants. It’s not what a [key stakeholder] thinks, it’s not what a health professional thinks what’s going to be the best.* (decision maker, health services)

#### Problem makers

Participants can also complicate effectiveness. For example, in one HIA a local councillor managed to stop the proposal in relation to which the HIA was being conducted. The other stakeholders in the HIA then re-scoped the HIA to focus on aspects contained within the original proposal, which could still be influenced.

*“There was a huge media beat up and as a [the two main organisations] withdrew from any future stages of the [name] strategy … but they both said, we’re going to take the recommendations from the HIA, they will go into council’s mainstream policies so they will inform – council’s still going to spend money on [name of area] and it’s not going to be called the [name] strategy…. [lead organisation] said to us that we will take their recommendations …..“(HIA working group, Health Sector)*

### Context factors

The institutions within which the stakeholders work and other broader contextual factors were identified as influencing effectiveness, including:

- **the planning and decision making context**
  *“There was a real commitment in the organisation to get it right and the organisation saw it as serious… it formed part of the development application documentation that needs to be assessed and signed off by local authorities, state authorities and then regional as well. It had to fit in with the context of regional locality.”* (decision-maker, local council)
- **broader global context**
  *“The other thing, is again a combination of what we were doing but also the, emerging national and international focus upon urban design and liveability and sustainability within cities. That was the context in which the [] Strategy was developed”. (decision-maker, regional organisation of councils)*
- **unanticipated events and activities that may influence a HIA**
  *“External factors that are out of your control. … So in between our starting and completing [the HIA], it [the land] was sold and we had to put the brakes on everything at one stage and that was precarious because it was then that [representative of decision-making organisation involved in HIA] left the region. We were quite nervous about that ‘cause it got handed to someone else in her team with less experience … it was an interesting fight for a while”. (HIA working group, health sector)*

Having shared values and goals between participants in the HIA was identified as positively influencing the perceived success of the HIA. These shared values can occur at a personal level (e.g. people directly involved in HIA process) and at organisational level (e.g. between organisations (inter-sectoral working) and between an organisation and the local community. It can facilitate trust between stakeholders and was seen by some to have been a powerful motivation for getting people engaged in the project, particularly where there had been a history of distrust.

*“Transparency, accountability, egalitarianism were inherent values there that we spoke about initially, those generic values to do with HIA but we really worked hard in trying, putting them into practice” (HIA working group, health sector)*

The effectiveness of HIAs is often judged by whether stated goals were met. This can be problematic when goals have not been made explicit.

*“it’s almost like you’re not allowed to sit around this table unless you can tick all these boxes and show you understand why you’re here because we’ve had people at the table that haven’t been properly briefed and they’ve come in with a traditional PPP [public private partnership], … contract management approach, it’s actually created a bit of conflict and you end up back tracking and it’s not good for progressing the partnership forward, you actually have to invest too much time on band aiding or sorting out those issues. I think values, alignment, commitment and understanding of the projects outcomes not the outputs”. (project proponent)*

Lack of clarity or agreement among stakeholders can lead to conflicting views on the success or effectiveness of the HIA. However having different purposes need not always be problematic. For example, in one case study one stakeholder focused on HIA capacity building, whereas another key stakeholder, who was also a decision-maker, wanted to improve their plan and planning process; yet the difference in purposes was not considered a problem. In this example although the purposes were different they were not in conflict with each other.

### Cross cutting themes

We also identified some factors that cut across the conceptual framework domains of context, process and impacts.

#### Proactive positioning

Respondents reported proactive engagement in the decision making cycle to either influence the cycle to fit the HIA or being flexible in the HIA process to fit the changing cycle as a key element of HIA effectiveness. For example, in one case it was recognised that the HIA was occurring too late in the decision making cycle to influence the strategy itself, so the team chose to re-scope the HIA to focus on an aspect of implementation which they could influence and was likely to have a significant impact on the local community.

*“At that stage it almost became derailed because we realised that our screening process may not have picked up the [timing]. It was after scoping that we actually realised that the HIA itself needed concentrating on the implementation of the policy [rather] than the actual decision making process …”* (HIA working group, health sector)

Another example describes how the HIA team pushed for the HIA to be included in the planning process.

“*So we thought, here’s an opportunity. We’ll see if they’ll be interested and will allow us to work with them to do the HIA … So there was negotiation with them, a lot of reassurance”. (HIA working group, health sector)*

Being in a proactive position is influenced by contextual factors such as organisational support and culture, existing processes and relationships that may sensitise individuals and organisations to recognise and act on opportunities to be involved in an HIA. This proactive positioning suggests that opportunities for HIA are more than just good timing.

“*The importance of being able to be flexible and at the same time then being able to meet the deadlines. …. The importance of ensuring the decision making was involved right through the process, the journey and the recommendations at the end and having ownership of that in effect. Those things were all very key”. (HIA working group, health sector)*

“*… where an organisation has taken a proactive lead and have someone inside the organisation who’s actually running it and doing it and writing it and we’re being brought in to be a support for the HIA, actually there's more buy-in and traction. And it has quite a long-term impact inside the organisation.” (HIA consultant)*

Having buy in and feelings of ownership by decision makers was perceived to increase the likelihood that recommendations were accepted and implemented.

A senior public servant described the actions they took to ensure that HIA was mainstreamed within the organisation in terms of formalising the organisation’s relationship with health.

“*… when I retire in a couple of years I guess from a succession planning perspective I wanted to make sure that the partnership was firmly entrenched in our business planning so that we know that when we start a new urban regeneration project the first tick off point is, let’s get HIA happening in some form”. (HIA commissioner, housing)*

#### Time and timeliness

Consistent with the HIA literature [16, 26–28], time, timing and timeliness were recurring themes in the eleven case studies [10] (see Table 3). Specifically the time, timing, and timeliness of the HIAs were often not ideal but that the process was flexible enough to be adapted to make it work. There was also no perfect time in the planning or decision-making cycle to carry out a HIA. In general earlier was seen to be better. Some HIAs were carried out late in the decision making process and even after decisions had been made but were still able to influence implementation and were perceived to be effective. Practitioners reported adapting the timing of the HIA during the process to maximise opportunities to influence decision-making.

**Table 3:** Components of time that influence HIA effectiveness

Time as a resource	Timeliness	Timing
Time to do the HIA	Wider drivers (e.g. interest in social determinants of health, built environment)	Timing the HIA in the planning cycle
Time to train to do the HIA	Fitting into existing work	Timing to influence decisions
Time to build and maintain relationships	People available	
Time to deal with changing circumstances	Multiple facilitators coinciding/streams aligning [36] (e.g. fed into strategic directions of the organisation, able to use new ways of thinking about the issue, experienced staff in place to take up opportunities)	
Ring-fencing time (dedicated time to work on HIA; organisational support to spend time on HIA)

*“I thought the timing was really good. I thought actually having [the proposal] before it was drafted and while it was still being written was a much more proactive place to start from”. (HIA consultant)*

*“The timing of this one I think was very good, it commenced after we had something on the table, but not a final proposal that was a done deal.”(decision maker, local council)*

Time was described as a resource: it was needed to carry out the HIA; to train to do the HIA; to build and maintain relationships; and to deal with changing circumstances. The value of having organisational support for dedicating specific personnel time to working on the HIA was emphasised.

*“I actually went off work for two weeks solid, I stopped my other role to do it, rather than doing what I did and trying to doing something else, so there was a real commitment in the organisation to get it right and the organisation saw it as serious”. (decision maker, local council)*

Factors identified as influencing the timeliness of HIAs ranged from wider drivers such as current interest in the relationship between the built environment and health to practical local factors such as the HIA fitting into existing workloads.

*“The other thing is again a combination of what we were doing but also the emerging or at that time - national and international focus upon urban design and liveability and sustainability within cities. That was coming through the media and so that was the context on which the Strategy was developed” (HIA working group, Health sector)*

*“there’s no good or bad time for the HIA …. We’ve already got the base data there to assist, it may pick up additional information we need to feed into that register; this list just helps galvanise and then work through it. Generally we’ve had the HIA’s completed prior to major works in an area so we can then strategically arrange work around what’s going in”. (HIA working group, engineering)*

## Discussion

We found multiple perspectives on what HIA effectiveness is, the effectiveness of specific HIAs, and the salience of different factors in influencing HIA effectiveness. However there were some features of the HIA process and contextual factors that were consistently identified as influencing the perceived effectiveness of HIAs.

Many of the factors identified as influencing effectiveness are consistent with HIA guidance documents and policy making literature e.g. [29, 30]. For example, having shared values and explicit goals was considered important, as was the involvement of decision-makers in the process, inter and intra-sectoral involvement, and relationships and partnerships between professional and community stakeholders. Some factors such as timing were, however, not as straight forward as they are often represented in the HIA literature. For example, HIA is often described as being best carried out early (but not too early) in the decision-making cycle [5, 31], we found examples of HIAs successfully influencing decision-making at various points in the decision-making processes – both early and late. Another example is having shared goals, we found that having a shared understanding of goals was important but that also organisational partners having different goals was not necessarily problematic where they tended to complement each other.

We found that in effective HIAs there is an aggregation of factors that contribute to effectiveness. We identified cross-cutting elements at different levels, e.g. timing and timeliness are important across every domain. In addition, factors influencing effectiveness can operate at different levels (e.g. individual, organisational and broader context), and proactive activity is both individual and institutional. All these factors need be viewed as cumulatively influencing decision making rather than as single factors in a check list which have to be gotten right.

Some factors that are important to HIA effectiveness are specific to the HIA process, some are features of the context and some are specific to the people involved. Some factors are necessary features of HIA (stepwise process, flexible, evidence based). Some are not essential features of HIA but can enhance the impact (proactive positioning, right person right level, relationships, partnerships, involvement of decision-makers, community, inter and intra-sectoral). Then there are the broader contextual factors outside of the HIA (planning and decision making context, broader global context, and unanticipated events and activities) that may influence a HIA. The range of policies, projects and plans on which HIA focus occur in complex open systems, which typically involve multiple decision-makers, multiple levels of decision making and multiple points of influence. By systems we mean psychologically, socially, and/or culturally defined entities and relations, which can include, for example, community, organisational, social, political, and regulatory systems. HIA processes can influence and are influenced by the systems within which they occur. These factors can be viewed through micro (individual), meso (institutional) and macro (broader context) lenses. The role and importance of these factors can change before, during and after the HIA.

When considering factors identified as influencing HIA effectiveness, a confluence of factors that come together to initiate a HIA are often identified (e.g. recent interest in healthy urban design, proposal for a new spatial plan, time was available, opportunity was recognised, the right person was available, it fitted into existing work, funding was available etc.). This can give the impression that HIAs are serendipitous in both their initiation and effectiveness. However, our interviews revealed that individuals and organisations actively took advantage of opportunities. We identified a meta-concept ‘proactive positioning’, which is linked to organisational and personal capacity to create and act on opportunities - HIA organisations and individuals need to be proactively positioned to recognise windows of opportunity or proactively create opportunity for HIA.

**Fig. 2:**
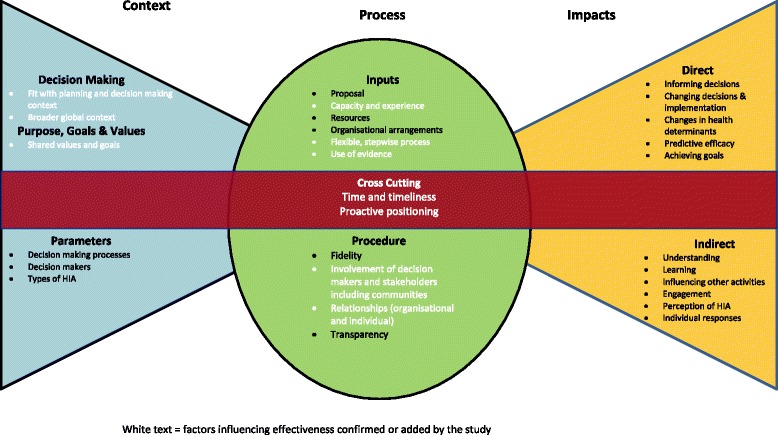
Revised framework for evaluating impact and effectiveness of HIA

These findings largely confirmed the conceptual framework by Harris-Roxas although some new influences were found or more clearly articulated. The framework provides a structured approach to considering and capturing both factors influencing HIA effectiveness and the outcomes that constitute effectiveness. However as the Harris-Roxas framework currently stands it is not able to capture cross cutting factors such as time and does not distinguish between different levels (e.g. individual versus organisational). We also identified some factors that were either not captured or were unclear within the framework (flexibility, relationships/partnerships, proactive positioning, time, timeliness and timing) (Fig. 2).

### Limitations

This research project has a number of limitations. Our sample is geographically specific to the New Zealand and Australian context, which may be different to that in other countries. We relied on participants’ perceptions, recall, and understanding of HIA effectiveness. Generalising our findings may be limited by other characteristics of our sample. The HIAs selected were often carried out by inexperienced practitioners and tended to be decision support HIAs (as opposed to mandated, advocacy or community led [15]). A large proportion (40 %) of the HIAs focused on land use. Finally, there is likely to be a tendency for less successful HIA processes not to be reported or even completed. Therefore although our sample showed a range of factors leading to HIA effectiveness, the sample was biased towards ‘the winners’. It was limited to 55 HIAs in phase one and then to 11 detailed HIA case studies. Two of the case studies were incomplete. Although additional data could strengthen our findings, we did collect a significant amount of data and reached a point of data saturation in our analysis. Future research should continue to identify case studies demonstrating the linkages between HIA recommendations and subsequent decisions.

## Conclusions

In this research project we sought to identify, disentangle and describe factors affecting HIA effectiveness. We went beyond existing frameworks, which identify factors for HIA effectiveness but do not provide a clear understanding of the most important factors and the relationships between them [19].

Identifying factors that influence HIA effectiveness, can inform and strengthen HIA practice. Much of what the study found is supported by previous research [19, 26, 27] and we have added depth to our understanding of how effectiveness can be improved. Our findings challenge some presumptions about the rationality and linearity of decision-making processes that are often underlying assumptions in HIA guides. In HIA there is often no one decision, no one decision maker, no ideal point in time or a linear (or even cyclical) decision-making process to influence. Instead there are multiple decisions to be potentially influenced and decisions that can be influenced by the findings of an HIA are often carried out at multiple points of time during the development and implementation of a proposal. There are also often a variety of different decision making agencies and individuals that can be influenced by the findings.

This research can help HIA practitioners and researchers understand what can be changed within the HIA process as well as determining external factors that may be outside of this process but have a significant impact on it. Paradoxically, an awareness of these ‘outside of control’ factors potentially gives practitioners more control through:

- where possible, adapting the flexible HIA process to accommodate such factors
- recognising policy windows and linking to actions outside of the HIA process (e.g. linking HIA into Health in All Policies approaches [32, 33]), or
- at the very least understanding and explaining why the HIA is currently not able to influence decision making.

Based on our findings, we recommend that HIA practitioners:

- invest time at the beginning of the HIA process discussing and clarifying purposes, goals, values and expected outcomes
- identify the relevant stakeholders and points of influence within systems and consider how the HIA can affect these
- involve stakeholders such as decision makers, people with knowledge about and access to decision making processes and also people with relevant skills as early as possible
- ensure HIA processes include potentially affected communities and build communities’ capacity and ability to engage in HIAs and decision-making processes
- position themselves proactively to recognise windows of opportunity and utilise the flexible but structured HIA process to adapt to these opportunities

If HIA is to become routine in the already complex set of planning and assessment processes currently adopted by both government and the private sector, within a context where HIA is often not a mandatory requirement, decision-makers will need to be convinced of its value. Given the growing popularity of HIA as an assessment tool, it also needs to be supported by a strong evidence both to validate its effectiveness and to make its application more robust. Whilst our research contributes to this evidence, future research should:

- investigate the completeness of the explanatory framework (are there important factors that are currently not included);
- be open to evaluating effectiveness of HIA beyond case studies to look at cost-effectiveness and impact of building HIA into other assessment processes, such as EIA;
- investigate differences between typologies of HIA [15];
- test whether the framework can help HIA stakeholders understand and manage the HIA process more effectively.

## Additional file

Additional file 1: Effectiveness of HIA studies (DOCX 21 kb)

## Abbreviation

HIA: Health impact assessment
