# Supplementary material for: What makes health impact assessments successful? Factors contributing to effectiveness in Australia and New Zealand
Source: BMC Public Health. 2015 Oct 3;15:1009. doi: 10.1186/s12889-015-2319-8 (PMC4592749; doi:10.1186/s12889-015-2319-8)
Supplement: Additional file 1: — Effectiveness of HIA studies (DOCX 21 kb) [file 12889_2015_2319_MOESM1_ESM.docx]

Additional File

| **Study** | **Country** | **Number of HIAs included** | **Sources of data** | **Main findings** |
| --- | --- | --- | --- | --- |
| Bekker, 2007  [1] | Netherlands | 3 and 2 simulated HIAs | Reports, interviews, game simulation | Found that a technical approach to HIA can be at odds with the political and administrative requirements that would make HIA work  HIAs can help to reframe policy issues  HIA may be most useful when considered a “coordination tool” for intersectoral work rather than a scientific approach |
| Bourcier, 2015  [2] | USA | 23 | Interviews, surveys, document review | Found HIAs can influence decisions in nonhealth-related sectors, influence changes beyond the decision target, build consensus and relationships among decision makers and their constituents, and give community members a stronger voice in decisions.  Found factors to increase HIA success include care in choosing a project or policy to be examined; selecting an appropriate team to conduct the HIA; engaging stakeholders and decision makers throughout the process; crafting clear, actionable recommendations; delivering timely, compelling messages to appropriate audiences; and using multiple dissemination methods. |
| Harris, Haigh et al,. 2013  [3] | Australia and New Zealand | 55 in document review, 44 in survey, 11 detailed case studies | Document review, survey, interviews, detailed case studies | Found all HIAs were effective in some way  Found HIAs brought about direct and indirect changes to decisions and implementation  Found timing, recognition of opportunities, having the right people involved, alignment with existing work and resourcing were related factors enhancing the impacts of HIAs  Found that HIAs led to increased skills and knowledge, conceptual learning and social learning. |
| Dannenberg, Bhatia et al., 2008  [4] | USA | 27 | Document review | Identified the characteristics of HIAs: type of proposal, HIA methods, nature of recommendations  Provides an overview of HIA practice in the US during 1999-2007 |
| O'Mullane, 2013  [5] | Ireland | 4 | Document review, 48 interviews | Willingness to work with external partners enabled HIAs to proceed  Broader contextual factors had a direct influence on the way the HIAs were conducted and received, e.g. government review of public administration  HIAs enabled subsequent related health promotion and planning activities |
| Opinion Leader Research, 2003  [6] | UK | 4 | Group discussion, observation, surveys | Found regulatory requirements facilitated the impact of the HIAs  Found mixed perceptions of HIA’s value amongst those involved in the HIAs  Lack of clarity about the extent to which any changes could be attributed to the HIAs |
| O'Reilly, Trueman et al., 2006  [7] | UK | 15 | Interviews, timesheets, surveys, willingness to pay surveys | HIAs enhanced the consideration of health impacts in decision-making  HIA was assigned a high monetary value by those involved in the process  It was difficult to attribute changes to the proposal to HIAs |
| Rhodus, Fulk et al., 2013  [8] | USA | 81 | Document review | Reviewed HIA attributes and level of rigour documented in reports  Relatively little information on changes to decision-making or implementation due to reliance on documentation  Attempted to categorise HIAs using Wismar’s effectiveness framework |
| Ward, 2006  [9] | New Zealand | 3 formally, 2 others informed report | Document review, interviews | HIA introduced new information to policy processes  Was a useful process for engaging stakeholders  Improved understanding of other agencies’ roles and activities  Important learning experience for those involved |

1. Bekker M: **The Politics of Healthy Policies: Redesigning Health Impact Assessment to Integrate Health in Public Policy**. Amsterdam: Eburon Delft; 2007.

2. Bourcier E, Charbonneau D, Cahill C, Dannenberg AL: **An Evaluation of Health Impact Assessments in the United States, 2011–2014**. *Preventing Chronic Disease* 2015, **12**.

3. Harris E, Haigh F, Baum F, Harris-Roxas B, Kemp L, Ng Chok H, Spickett J, Keleher H, Morgan R, Harris M *et al*: **The Effectiveness of Health Impact Assessment in New Zealand and Australia 2005-2009**. Sydney: Centre for Primary Health Care and Equity, University of New South Wales; 2013.

4. Dannenberg AL, Bhatia R, Cole BL, Heaton SK, Feldman JD, Rutt CD: **Use of Health Impact Assessment in the U.S: 27 Case Studies, 1999-2007**. *American Journal of Preventive Medicine* 2008, **34**(3):241-256.

5. O'Mullane M, Quinlivan A: **Health Impact Assessment (HIA) in Ireland and the role of local government**. *Environmental Impact Assessment Review* 2012, **32**(1):181-186.

6. Opinion Leader Research: **Report on the Qualitative Evaluation of Four HIAs on Draft Mayoral Strategies for London**; 2003.

7. O'Reilly J, Trueman P, Redmond S, Yi Y, Wright D: **Cost Benefit Analysis of Health Impact Assessment**; 2006.

8. Rhodus J, Fulk F, Autrey B, O’Shea S, Roth A: **A Review of Health Impact Assessments in the U.S.: Current State-of-Science, Best Practices, and Areas for Improvement**. In*.* Cincinnati: Office of Research and Development, National Exposure Research Laboratory, U.S. Environmental Protection Agency; 2013.

9. Ward M: **Health Impact Assessment in New Zealand: Experience at policy level**; 2006.
